# Exploration of the treatment challenges in men with intellectual difficulties and testicular cancer as seen in Down syndrome: single centre experience

**DOI:** 10.1186/s12916-015-0386-4

**Published:** 2015-06-26

**Authors:** Shaista Hafeez, Mausam Singhera, Robert Huddart

**Affiliations:** The Institute of Cancer Research, 123 Old Brompton Road, London, SW7 3RP UK; The Royal Marsden NHS Foundation Trust, Downs Road, Sutton, Surrey SM2 5PT UK; Guy’s and St Thomas’ NHS Foundation Trust, St Thomas Hospital, Westminster Bridge Road, London, SE1 7EH UK

**Keywords:** Chemotherapy, Down syndrome, Radiotherapy, Testicular cancer, Trisomy 21

## Abstract

Down syndrome is the most common chromosomal disorder in humans as well as the most common cause of inherited intellectual disability. A spectrum of physical and functional disability is associated with the syndrome as well as a predisposition to developing particular malignancies, including testicular cancers. These tumours ordinarily have a high cure rate even in widely disseminated disease. However, individuals with Down syndrome may have learning difficulties, behavioural problems, and multiple systemic complications that have the potential to make standard treatment more risky and necessitates individualized approach in order to avoid unacceptable harm. There is also suggestion that tumours may have a different natural history. Further, people with learning disabilities have often experienced poorer healthcare than the general population. In order to address these inequalities, legislation, professional bodies, and charities provide guidance; however, ultimately, consideration of the person in the context of their own psychosocial issues, comorbidities, and possible treatment strategies is vital in delivering optimal care. We aim to present a review of our own experience of delivering individualized care to this group of patients in order to close the existing health inequality gap.

## Background

Germ cell tumours make up 95 % of malignant tumours of the testes. Although rare (constituting 2 % of all human malignancies), they are the most common solid tumours in young men between the ages of 15 and 44 years [1]. Sensitivity to both chemotherapy and radiotherapy lends itself to excellent cure rates, even in the metastatic setting, with 10-year survival of 96 % across all stages [1]. Deviation from standard care can significantly compromise outcomes [1]. Further, the predisposition to testicular cancer in those with Down syndrome raises particular challenges with respect to this [3, 12].

The Royal Marsden NHS Foundation Trust acts as a supraregional centre and manages all advanced germ cell tumours or cases requiring complex surgical or medical management for a population of 5.6 million people in South East England, seeing over 100 new patients a year. We have previously presented our case series of Down syndrome and testicular germ cell tumours over a 20-year period [2]. Here, we aim to review the considerations necessary when treating this group of patients and present important aspects to support individual physical and psychological needs to deliver best care from diagnosis to treatment and subsequent long-term follow-up.

## Methods

A search was made of our personal referencing archives relating to the epidemiology, diagnosis, and management of testicular germ cell cancers and Down syndrome. The Cochrane Database was also interrogated for systematic reviews or meta-analyses. We also reviewed guidelines published by the European Society of Medical Oncology, the European Germ Cell Cancer Collaborative Group, and national and international public health bodies regarding best care of those with learning or intellectual disability. This paper reflects expert opinion and current literature accessed by the authors; no other formal search strategy has been defined.

### Clinical features of Down syndrome

Down syndrome is the most common and best-known chromosomal disorder in humans; 95 % of cases are due to trisomy of chromosome 21. This genetic imbalance gives rise to multiple systemic complications, including increased susceptibility to respiratory infections, congenital cardiac abnormality (up to 60 % of children), gastrointestinal abnormalities such as gastro-intestinal atresia and Hirschsprung’s disease, orthopaedic complications, thyroid disorders, hearing and visual impairment, and urological abnormalities including hypospadias, cryptorchidism (up to 27 % of males), and renal malformations (3.5 %) [3, 4]. There is also a predisposition to other cancers, including leukaemia, of which there is a 10- to 20-fold increased risk [5, 6].

Down syndrome is also the most common cause of inherited intellectual disability and accounts for one third of all moderate and severe learning difficulties in children [7]. Almost all individuals with Down syndrome demonstrate some degree of cognitive impairment. The spectrum of deficit is wide; most have an IQ in the 50 to 70 or 35 to 50 range, consistent with mild to moderate intellectual disability respectively, whereas some have severe impairment with an IQ in the 20 to 35 range [8]. Cognitive function is also affected by early onset dementia, with Alzheimer-like changes evident in >50 % of those over the age of 35 years [4, 9]. Compared to the general population, they experience greater neurophysical morbidity, including epilepsy, autism, behavioural difficulties, and mood disorders [4]. As a result, individuals are less likely to recognise or communicate that they are experiencing a problem.

Although the phenotypic manifestation of Down syndrome varies between individuals, the combination of learning disability and co-morbidity can often lead to non-standard treatment for many disorders. Given testicular cancer has a high cure rate even in the presence of metastatic disease, the risk of non-standard oncological treatment for those with Down syndrome raises the likelihood of treatment failure [1]. These tumours may also have an altered natural history that may in turn effect cure [10–12].

It can be hypothesized that the cytogenetic aberrations seen in Down syndrome could lead to altered biological features in germ cell tumours; this possibility is exemplified in acute lymphoblastic leukaemia (ALL). The immunophenotypic and biological features of ALL in those with Down syndrome are distinctly different from those without [13].

When outcomes of ALL in 653 children with Down syndrome enrolled in 16 international trials from 1995 to 2004 were examined and compared to 4445 children with ALL and no Down syndrome, it was found that Down syndrome-associated ALL had a significantly higher 8-year cumulative incidence of relapse (26 % vs 15 %), poorer event-free 8-year survival (64 % vs 81 %), and inferior overall survival at 8 years (74 % vs 89 %). In addition, they experienced significantly worse treatment-related mortality independent of therapeutic regime (7 % vs 2 %), with the most common cause of death resulting from infection [14]. The small sample size of reported Down syndrome and testicular cancer series has precluded definitive conclusions to be drawn regarding similar outcome parameters [2, 15].

### Incidence and aetiology of testicular cancers in Down syndrome

Testicular germ cell cancers affect mainly young men, with 85 % presenting between 15 and 44 years of age [1]. Germ cell tumours are classified as pure seminomas or non-seminomatous germ cell tumours, which include variants such as embryonal carcinoma, teratocarcinoma, yolk sac tumour, choriocarcinoma, and teratoma [16]. The incidence of pure seminomas peaks in men aged 30 to 45 years, whereas non-seminomatous germ cell tumours (NSGCTs) peak in men aged 20 to 35 years [17]. In Down syndrome, most testicular tumours are seminomas irrespective of age [15].

Although there is global variation, the incidence of testicular germ cell tumours in the general population of Western Europe is 0.09 % compared to 0.5 % in those with Down syndrome [12, 15, 18, 19]. The exact biological mechanism for this predisposition is unknown. Contributing factors are thought to include cryptorchidism, high levels of gonadotropins especially follicle-stimulating hormone, an oncogenic gene dose effect due to the extra genetic material present, delayed germ cell maturation in the fetus, increased sensitivity of trisomic cells to carcinogens, or germ cell oncogenesis in utero occurring with increased maternal age [4, 5, 12, 20–22].

### Presentation of testicular cancer in Down syndrome compared to the general population

Ordinarily, more than 95 % of men with testicular cancer will present to the urologist with a lump in testis that is often painless [23]. In patients with Down syndrome, diagnosis is usually as a result of an incidental finding. More than half of the cases are detected during in-patient hospital treatment for other causes, or by the primary care giver, with self-reporting least likely to lead to diagnosis [2].

To improve patient awareness and guide self-examination a number of easy-read picture booklets have been produced. In view of the potential communication issues, increased vigilance for those providing routine clinical care to those with Down syndrome should be encouraged with testicular examination performed where appropriate during routine health checks [4].

In view of difficulties in self-reporting, it is often assumed that these tumours present later and at a more advanced stage. However, there is no data to support this, and stage of presentation is often comparable with that of the general population [12, 24].

### Communication, informed consent, and patient-centred decision-making challenges in Down syndrome

People with learning disabilities often experience poorer healthcare than the general population and significant failings have been identified [25]. In response to this, key reports (in the UK) have been commissioned to bring about change to the way care is delivered and outline what reasonable adjustments need to be made in order to ensure there is equity [26–28]. As is the case throughout the world, legislation is often needed to ensure these rights are maintained. Important legislation in the UK includes the Mental Capacity Act 2005, the Disability Discrimination Act 2005, and the Human Rights Act 1998, all of which inform the work of professionals caring for those with learning difficulties [29, 30]. The Mental Capacity Act, in particular, covers a number of important aspects, including introducing an independent Mental Capacity Advocate Service and establishing a code of practice and a framework for delivering care to those who may lack capacity [31].

The degree of learning difficulties exhibited in our previously reported patient group meant no individual had the capacity to give informed consent about their care and therefore a decision based on the patients’ best interest was made [2]. Best interest decisions are guided by what the individual wants, the appointed advocate, and by listening to those who know and care for the individual. In the absence of a best interest consensus, in the UK, a judicial decision would then be made by the Court of Protection [31]. The Mental Capacity Act also clarifies on the legality of enrolling individuals in certain closely regulated medical research. Enrolment in trials is permitted with strict safeguards to ensure that the research is expected to benefit the individual directly and that the risks are not excessive in relation to the anticipated benefits. Before enrolment, researchers must also identify somebody close to the individual or, in their absence, someone independent to the researchers who is willing to be consulted about the appropriateness of their involvement [31].

Non-governmental organizations are also an important resource for healthcare professionals to guide patient-centred care. The UK charity for those with learning disability, *Mencap*, has recommended key issues to ensure best practice is maintained [32], including longer appointment times, communication with the individual (verbal and non-verbal), listening to the knowledge of families and carers, and valuing the life of the person with a learning disability.

Poor communication in many instances has led to shortcomings in care delivery [25]. However, there are many ways in which communication can be aided and information made more accessible, including easy-read information, picture guides, and individualised health plans. Many institutions also have a variation on the ‘patient passport’, which ensures that during their care, patients are presented as individuals and not merely as a sum of their diagnoses. It contains information on particular likes, dislikes, and needs in order to facilitate successful interactions [32, 33]. It is also important to recognise that those with profound and multiple learning disabilities may not communicate formally with speech or pictures but use facial expressions, vocal sounds, and body language. There are different techniques that can be used to help interpret their needs in order to facilitate care. Often, those who support and care for these individuals have spent time getting to know their means of communication and have found effective personal means of interaction and are therefore an important resource [32].

There are many complex issues facing people with learning difficulties and their access to healthcare, but central to almost all failings is often the lack of value placed on the life of someone with a learning disability [25, 34]. A number of specific factors and systematic shortcomings have been identified [35, 36]. The Confidential Injury into Premature Deaths of People with Intellectual Disabilities (CIPOLD) investigated all known events leading to the death of 247 people with intellectual disability over a 2-year period. The causes identified in a subset of those with intellectual disabilities included problems with advanced care planning, adherence to the Mental Capacity Act, inappropriate accommodation, not adjusting care as needs changed, and carers not feeling listened to [37]. In addition, they experienced significantly more problems with diagnosis and treatment, as well as with all other aspects of care provision, planning, coordination, and documentation. Alarmingly, 37 % of all deaths of people with intellectual disabilities were found to be avoidable and from causes that were potentially amenable to change by better quality healthcare [37].

The presence of discrimination, abuse, and neglect across the range of health services is well documented and we would recommend review of these reports to ensure that any intentional or unintentional negative assumptions are guarded against in order to prevent avoidable harm [34, 35, 37]. General staff training to improve understanding and awareness of reasonable adjustments and patient needs will help improve patient safety and outcomes [35].

### Treatment challenges of germ cell tumours compared to the general population

At the time of initial presentation most men presenting with testicular cancer undergo a radical inguinal orchidectomy for both diagnosis and definitive treatment. For men presenting with advanced disease, radical orchidectomy is preferred prior to chemotherapy where possible except in circumstances of life threatening advanced disease when pathological diagnosis is made from metastatic lesion biopsy and orchidectomy may be delayed until after the completion of chemotherapy. The pathology informs treatment stratification as seminomas and NSGCT differ in biology. In general, seminomas are less likely to metastasise to viscera (men commonly present with localised disease – 80 % will have stage I disease at presentation), they are more sensitive to radiotherapy treatment than NSGCTs, and are not usually associated with elevated serum tumour markers (beta-human chorionic gonadotropin and alpha fetoprotein) [1]. An overview of the standard management of testicular germ cell tumours in the general population can be found through a number of sources [1, 38].

We have previously reported on the medical management of patients with histologically confirmed testicular cancer and Down syndrome treated at the Royal Marsden Foundation Trust between 1982 and 2005 and found that their known comorbidities has an impact on conventional treatment delivery [2]. Prior to starting therapy, ordinarily cryopreservation is made available to all men diagnosed with testicular cancer should they wish to preserve fertility [1]. Almost all men with Down syndrome are infertile because of impaired spermatogenesis [39]. There have, however, been reported cases of live births fathered by men with Down syndrome [40]. Given that the impact of Down syndrome on each person is variable, with some able to live independently as adults, assumptions and expectations about fertility should be explored on a case-by-case basis.

### Management of seminoma and deviations from standard care

In general, stage I seminoma can be managed with surveillance following orchidectomy with an anticipated relapse rate of 18 % [41]. Surveillance would be our general population-based recommendation for those with one or no risk factors for relapse, as most will be successfully treated with no adverse impact on oncological outcome. It is reliant, however, on compliance and long-term commitment to attend for regular follow-up as time to relapse can be prolonged [1]. The intra-abdominal nature and predominate marker negative status of relapsed disease requires regular cross sectional imaging [1]. The potential distress and emotional impact this may cause to those with Down syndrome and intellectual disabilities despite appropriate support should be judged individually.

Alternatively, adjuvant carboplatin or radiotherapy can be considered in all patients who decline surveillance, reducing the risk of relapse to less than 5 % [42–44]. It is also considered in those who are less able to comply or commit to the regular follow-up surveillance requires. Adjuvant treatment is often considered in those with Down syndrome to spare more intense combination chemotherapy at disease relapse, which may be poorly tolerated. Despite this approach, relapse rates appear as high as 75 % in Down syndrome, still necessitating further combination chemotherapy [2]. Although the patient numbers are small, the high relapse rate despite adequate initial treatment supports previous evidence of altered tumour biology compared to the general population [12].

Following orchiectomy, the optimal treatment for stage II seminoma conventionally depends upon whether there is bulky lymph node involvement [1]. In low volume stage II seminoma, radiotherapy to the para-aortic and ipsilateral lymph node region is an option. We have combined initial single cycle carboplatin with subsequent lower dose radiotherapy to the para-aortic nodes alone to mitigate side effects and long-term toxicity with the larger radiation fields and higher dose as our conventional standard [45]. Multi-agent chemotherapy (BEP; bleomycin, etopside, and cisplatin) is used in bulkier and more advanced disease. However, deviation from standard combination cisplatin-based chemotherapy is often found to be necessary in those with Down syndrome to avoid potential significant toxicity [2]. In particular, substitution of cisplatin is made with carboplatin both because of renal impairment and in order to reduce fluid load in those with pre-existing cardiac malformations. Although 3 to 4 cycles of single agent carboplatin may appear attractive in terms of toxicity profile, it is associated with higher rates of failure and so should only be considered in circumstances where the risk of standard multi-agent chemotherapy significantly outweighs the benefit [46].

The expected relapse rate for stage II disease following standard therapy is 10 % [47]. In those with Down syndrome and stage II disease, treated with four cycles of carboplatin, more than 30 % of patients fail treatment (although death from disease remains rare) [2]. Despite drug modifications, patients are still likely to suffer chemotherapy toxicity. Over half of patients require hospital admission for complications on treatment, including sepsis, pulmonary oedema, and seizures [2]. This high failure rate and poor tolerance supports the hypothesis that unique biological factors affect treatment outcome, as is seen in those with ALL [14, 48]. Therefore, given the poor tolerance of chemotherapy, in stage IIa/IIb seminoma, radiotherapy should be considered an important alternative radical treatment option [16, 49].

### Management of non-seminomatous germ cell tumours (NSGCTs) and deviations from standard care

NSGCTs are less common in those with Down syndrome than the general testicular cancer patient population. In stage I disease conventionally following orchidectomy 30 % of all men will relapse with recurrent disease and 50 % will relapse if lymphovascular invasion was present on pathology [1]. The majority (95 %) of these relapses will occur within the first 2 years; on relapse, cure is close to 100 % with chemotherapy [1, 50]. Therefore, although surveillance following orchiectomy is acceptable in the general population, the individual appropriateness of an intense surveillance protocol should be judged and discussed with the patient and carers in Down syndrome as poor adherence to surveillance could mean potential recurrence at advanced stage disease necessitating more chemotherapy than would have been required in the adjuvant setting.

The efficacy of two cycles of adjuvant BEP is established in routine care, but there is accumulating evidence that a single cycle maybe equally as effective and so may be preferable when poor tolerance of chemotherapy is anticipated [50, 51]. Retroperitoneal lymph node dissection may also be an option in the adjuvant setting in those unsafe/unsuitable for chemotherapy, although it is known to be inferior to one cycle of BEP [51]. In the metastatic setting, three or four cycles of BEP chemotherapy would be considered standard depending on the International Germ Cell Cancer Collaborative Group risk classification, but careful assessment of fitness for safe administration of this regime would be required [1].

We have found that learning disability in itself does not and should not preclude radical therapy. However, in the presence of moderate and severe learning disabilities, general anaesthetic is occasionally necessary in order to aid compliance to investigations, complete diagnostic staging, and safely deliver radiotherapy. Importantly, due to the known behavioural disorders, lack of cooperation should not be assumed to be necessarily associated with a lack of consent and other factors, including distress, should be considered.

### Importance of recognising treatment-related psychological distress

A cancer diagnosis can lead to psychological distress in up to 75 % of cases in the general cancer patient population [52]. In view of this, current UK government policy recommends that all patients should undergo systematic psychological assessment at key points during their cancer journey complimented by access to appropriate psychological support services. There is, however, a lack of clarity about the most effective way to address this psychological distress. Nurse-delivered interventions combining information with supportive attention may have a beneficial impact on mood in an undifferentiated population of newly diagnosed cancer patients [52]. A randomized control trial has shown no evidence of benefit for routine psychological therapy in newly diagnosed patients with testicular cancer [53]. However, in line with Moynihan et al. [53], we would suggest that, although specific interventions must be systematically evaluated, informing and reassuring patients should be seen as an integral part of routine care.

Behavioural and psychiatric disorders are common in Down syndrome, with an estimated 22 % of individuals affected (18 % under the age of 20) [4, 54]. The most common manifestation is usually disruptive, aggressive and repetitive behaviours, anxiety disorders, and major depressive illnesses [54]. This pre-existing psychological distress can be exacerbated by the diagnosis of testicular cancer and the change in usual environment and routine that subsequent treatment entails. Deterioration in family social interaction as a result of repeated hospitalisation due to both chemotherapy and the complications arising from it is seen [2], highlighting the importance of recognising worsening mental health problems and offering appropriate support to deliver holistic care.

### Survivorship issues

The successful management of germ cell tumours in the general population has meant that emphasis is now on minimising treatment-related toxicity, ensuring appropriate follow-up mechanisms are in place to detect relapse, addressing long term treatment side effects, and effective rehabilitation. These survivorship aims are now also increasingly pertinent to those with Down syndrome given survival in Down syndrome has improved substantially in recent years and is approaching that of the general population, owed predominantly to improved management of congenital cardiac abnormalities [55]. Death from cancer in Down syndrome remains rare. The most common contributing causes of death are usually pneumonias, other infections, congenital malformations, circulatory disease, and dementia [56, 57].

The young age at presentation and high cure rates of testicular cancer in the general population, mean that it becomes increasingly important to recognise and minimise long-term treatment toxicity. The most common potentially life-threatening late effects, usually occurring more than 10 years after treatment, are second malignancy and cardiovascular disease [58, 59]. Other long-term effects include pulmonary toxicity, nephrotoxicity, neurotoxicity, decreased fertility, hypogonadism, and psychosocial problems [58]. Given the improved survival, similar consideration should also be made to the long-term health consequences in Down syndrome testicular cancer survivors. Individuals with Down syndrome have a reduced resting metabolic rate, which contributes to higher rates of obesity [4, 60]. This predisposition further increases the likelihood of metabolic syndrome, which has been shown to be higher after testicular cancer treatment and may be a significant contributing factor in the development of cardiovascular disease following treatment [61].

## Conclusions

Patients with testicular cancer and Down syndrome have complex physical and psychological needs. Their intellectual disability should not preclude radical treatment. Acknowledgement has been made by a number of reports of the potential ‘invisibility’ of this group of patients, so vigilance and procedure to guard against this needs to be in place to ensure best practice is maintained. The wide spectrum of comorbidities means an individualized approach is necessary and often requires modification of standard chemotherapy treatment in order to deliver safe care. Even when no deviation is made to standard care, there is suggestion of altered tumour biology and outcome compared to the general population that warrants further investigation. Despite this, a high cure rate can be achieved with appropriate therapy.

## Abbreviations

ALL: Acute lymphoblastic leukaemia
BEP: Bleomycin, etoposide, cisplatin combination therapy
NSGCT: Non-seminomatous germ cell tumours

## References

[1] Horwich A, Nicol D, Huddart R (2013). Testicular germ cell tumours. BMJ..

[2] Hafeez S, Sharma RA, Huddart RA, Dearnaley DP, Horwich A (2007). Challenges in treating patients with Down’s syndrome and testicular cancer with chemotherapy and radiotherapy: The Royal Marsden experience. Clin Oncol (R Coll Radiol)..

[3] Mercer ES, Broecker B, Smith EA, Kirsch AJ, Scherz HC, Massad AC (2004). Urological manifestations of Down syndrome. J Urol..

[4] Roizen NJ, Patterson D (2003). Down’s syndrome. Lancet..

[5] Wiseman FK, Alford KA, Tybulewicz VL, Fisher EM (2009). Down syndrome-recent progress and future prospects. Hum Mol Genet..

[6] Tabares-Seisdedos R, Dumont N, Baudot A, Valderas JM, Climent J, Valencia A (2011). No paradox, no progress: inverse cancer comorbidity in people with other complex diseases. Lancet Oncol..

[7] Rauch A, Hoyer J, Guth S, Zweier C, Kraus C, Becker C (2006). Diagnostic yield of various genetic approaches in patients with unexplained developmental delay or mental retardation. Am J Med Genetics Part A..

[8] Bull MJ, Committee on Genetics (2011). Health supervision for children with Down syndrome. Pediatrics..

[9] Lai F, Williams RS (1989). A prospective study of Alzheimer disease in Down syndrome. Arch Neurol..

[10] Hasle H, Clemmensen IH, Mikkelsen M (2000). Risks of leukaemia and solid tumours in individuals with Down’s syndrome. Lancet..

[11] Lange B (2000). The management of neoplastic disorders of haematopoiesis in children with Down’s syndrome. Br J Haematol..

[12] Hasle H (2001). Pattern of malignant disorders in individuals with Down’s syndrome. Lancet Oncol..

[13] Maloney KW (2011). Acute lymphoblastic leukaemia in children with Down syndrome: an updated review. Br J Haematol..

[14] Buitenkamp TD, Izraeli S, Zimmermann M, Forestier E, Heerema NA, van den Heuvel-Eibrink MM (2014). Acute lymphoblastic leukemia in children with Down syndrome: a retrospective analysis from the Ponte di Legno study group. Blood..

[15] Satge D, Sasco AJ, Cure H, Leduc B, Sommelet D, Vekemans MJ (1997). An excess of testicular germ cell tumors in Down’s syndrome: three case reports and a review of the literature. Cancer..

[16] Horwich A, Shipley J, Huddart R (2006). Testicular germ-cell cancer. Lancet..

[17] Singhera M, Huddart R (2013). Testicular cancer: changing patterns of incidence in testicular germ cell tumours. Nat Rev Urol..

[18] GLOBOCAN 2012. Estimated cancer incidence, mortality and prevalence worldwide. 2012. http://globocan.iarc.fr/.

[19] Satge D, Vekemans M (2011). Down syndrome patients are less likely to develop some (but not all) malignant solid tumours. Clin Genet..

[20] Smucker JD, Roth LM, Sutton GP, Hurteau JA (1999). Trisomy 21 associated with ovarian dysgerminoma. Gynecol Oncol..

[21] Cools M, Honecker F, Stoop H, Veltman JD, de Krijger RR, Steyerberg E (2006). Maturation delay of germ cells in fetuses with trisomy 21 results in increased risk for the development of testicular germ cell tumors. Hum Pathol..

[22] Forman D, Gallagher R, Moller H, Swerdlow TJ (1990). Aetiology and epidemiology of testicular cancer: report of consensus group. Prog Clin Biol Res..

[23] Trama A, Mallone S, Nicolai N, Necchi A, Schaapveld M, Gietema J (2012). Burden of testicular, paratesticular and extragonadal germ cell tumours in Europe. Eur J Cancer..

[24] Dieckmann KP, Rube C, Henke RP (1997). Association of Down’s syndrome and testicular cancer. J Urol..

[25] Mencap. Death by indifference. London: Mencap; 2007.

[26] Hatton C, Roberts H, Baines S. Reasonable adjustments for people with learning disabilities in England: a national survey of NHS Trusts. Improving Health and Lives: Learning Disabilities Observatory. 2011. http://www.improvinghealthandlives.org.uk/projects/reasonableadjustments.

[27] Giraud-Saunders A. Equal access? A practical guide for the NHS: creating a Single Equality Scheme that includes improving access for people with learning disabilities. Department of Health. 2009. www.ncuh.nhs.uk/patients-and-visitors/accessibility/equal-access.pdf.

[28] Turner S, Robinson C. Reasonable adjustments for people with learning disabilities: implications and actions for commissioners and providers of health care. Evidence into practice report no. 3. Improving Health and Lives: Learning Disabilities Observatory. 2011. http://www.improvinghealthandlives.org.uk/uploads/doc/vid_11084_IHAL%202011%20-01%20Reasonable%20adjustments%20guidance.pdf.

[29] Disability Discrimination Act (2005). London: HM Government.

[30] Mental Capacity Act 2005 (Loss of Capacity During Research Project) (England) Regulations 2007. London: HM Government.

[31] The Mental Capacity Act 2005. Guidance for health professionals. 2007. British Medical Association.. http://bma.org.uk/practical-support-at-work/ethics/mental-capacity/assessing-mental-capacity. Last accessed 11/6/15.

[32] Mencap. https://www.mencap.org.uk/about-learning-disability/information-professionals/health. Last accessed 11/6/15.

[33] The Royal Marsden. Learning disability services. http://www.royalmarsden.nhs.uk/diagnosis-treatment/patient-support/pages/learning-disability.aspx. Last accessed 11/6/15.

[34] Michael J (2008). Healthcare for all: report of the independent inquiry into access to healthcare for people with learning disabilities.

[35] Tuffrey-Wijne I, Goulding L, Gordon V, Abraham E, Giatras N, Edwards C (2014). The challenges in monitoring and preventing patient safety incidents for people with intellectual disabilities in NHS acute hospitals: evidence from a mixed-methods study. BMC Health Serv Res..

[36] Tuffrey-Wijne I, Goulding L, Giatras N, Abraham E, Gillard S, White S (2014). The barriers to and enablers of providing reasonably adjusted health services to people with intellectual disabilities in acute hospitals: evidence from a mixed-methods study. BMJ Open..

[37] Heslop P, Blair PS, Fleming P, Hoghton M, Marriott A, Russ L (2014). The confidential inquiry into premature deaths of people with intellectual disabilities in the UK: a population-based study. Lancet..

[38] Oldenburg J, Fossa SD, Nuver J, Heidenreich A, Schmoll HJ, Bokemeyer C (2013). Testicular seminoma and non-seminoma: ESMO Clinical Practice Guidelines for diagnosis, treatment and follow-up. Ann Oncol.

[39] Johannisson R, Gropp A, Winking H, Coerdt W, Rehder H, Schwinger E (1983). Down’s syndrome in the male. Reproductive pathology and meiotic studies. Hum Genet.

[40] Sheridan R, Llerena J, Matkins S, Debenham P, Cawood A, Bobrow M (1989). Fertility in a male with trisomy 21. J Med Genet..

[41] Cummins S, Yau T, Huddart R, Dearnaley D, Horwich A (2010). Surveillance in stage I seminoma patients: a long-term assessment. Eur Urol..

[42] Oliver RT, Mason MD, Mead GM, von der Maase H, Rustin GJ, Joffe JK (2005). Radiotherapy versus single-dose carboplatin in adjuvant treatment of stage I seminoma: a randomised trial. Lancet..

[43] Jones WG, Fossa SD, Mead GM, Roberts JT, Sokal M, Horwich A (2005). Randomized trial of 30 versus 20 Gy in the adjuvant treatment of stage I testicular seminoma: a report on Medical Research Council Trial TE18, European Organisation for the Research and Treatment of Cancer Trial 30942 (ISRCTN18525328). J Clin Oncol..

[44] Oliver RT, Mead GM, Rustin GJ, Joffe JK, Aass N, Coleman R (2011). Randomized trial of carboplatin versus radiotherapy for stage I seminoma: mature results on relapse and contralateral testis cancer rates in MRC TE19/EORTC 30982 study (ISRCTN27163214). J Clin Oncol..

[45] Gilbert DC, Van As NJ, Beesley S, Bloomfield D, Money-Kyrle J, Norman A (2009). Treating IIA/B seminoma with combination carboplatin and radiotherapy. J Clin Oncol..

[46] Krege S, Boergermann C, Baschek R, Hinke A, Pottek T, Kliesch S (2006). Single agent carboplatin for CS IIA/B testicular seminoma. A phase II study of the German Testicular Cancer Study Group (GTCSG). Ann Oncol.

[47] Warde P, Huddart R, Bolton D, Heidenreich A, Gilligan T, Fossa S (2011). Management of localized seminoma, stage I–II: SIU/ICUD Consensus Meeting on Germ Cell Tumors (GCT), Shanghai 2009. Urology..

[48] Patrick K, Wade R, Goulden N, Rowntree C, Hough R, Moorman AV (2014). Outcome of Down syndrome associated acute lymphoblastic leukaemia treated on a contemporary protocol. Br J Haematol..

[49] Chung P, Warde P (2013). Contemporary management of stage I and II seminoma. Curr Urol Rep..

[50] Cullen MH, Stenning SP, Parkinson MC, Fossa SD, Kaye SB, Horwich AH (1996). Short-course adjuvant chemotherapy in high-risk stage I nonseminomatous germ cell tumors of the testis: a Medical Research Council report. J Clin Oncol..

[51] Albers P, Siener R, Krege S, Schmelz HU, Dieckmann KP, Heidenreich A (2008). Randomized phase III trial comparing retroperitoneal lymph node dissection with one course of bleomycin and etoposide plus cisplatin chemotherapy in the adjuvant treatment of clinical stage I nonseminomatous testicular germ cell tumors: AUO trial AH 01/94 by the German Testicular Cancer Study Group. J Clin Oncol..

[52] Galway K, Black A, Cantwell M, Cardwell CR, Mills M, Donnelly M (2012). Psychosocial interventions to improve quality of life and emotional wellbeing for recently diagnosed cancer patients. Cochrane Database Syst Rev..

[53] Moynihan C, Bliss JM, Davidson J, Burchell L, Horwich A (1998). Evaluation of adjuvant psychological therapy in patients with testicular cancer: randomised controlled trial. BMJ..

[54] Myers BA, Pueschel SM (1991). Psychiatric disorders in persons with Down syndrome. J Nerv Ment Dis..

[55] Irving CA, Chaudhari MP (2012). Cardiovascular abnormalities in Down’s syndrome: spectrum, management and survival over 22 years. Arch Dis Child..

[56] Yang Q, Rasmussen SA, Friedman JM (2002). Mortality associated with Down’s syndrome in the USA from 1983 to 1997: a population-based study. Lancet..

[57] Englund A, Jonsson B, Zander CS, Gustafsson J, Anneren G (2013). Changes in mortality and causes of death in the Swedish Down syndrome population. Am J Med Genet A..

[58] Haugnes HS, Bosl GJ, Boer H, Gietema JA, Brydoy M, Oldenburg J (2012). Long-term and late effects of germ cell testicular cancer treatment and implications for follow-up. J Clin Oncol..

[59] Huddart RA (2003). Improving treatment outcomes in testicular cancer: strategies to reduce treatment related morbidity. BJU Int..

[60] Rubin SS, Rimmer JH, Chicoine B, Braddock D, McGuire DE (1998). Overweight prevalence in persons with Down syndrome. Ment Retard..

[61] Nuver J, Smit AJ, Wolffenbuttel BH, Sluiter WJ, Hoekstra HJ, Sleijfer DT (2005). The metabolic syndrome and disturbances in hormone levels in long-term survivors of disseminated testicular cancer. J Clin Oncol..

